# Pulmonary Arterial Hypertension in Genetic and Comorbid Settings: A Step Forward for Precision Medicine

**DOI:** 10.3390/jcm11226671

**Published:** 2022-11-10

**Authors:** Rosalinda Madonna

**Affiliations:** Cardiology Division, Institute of Cardiology, University of Pisa, C/o Ospedale di Cisanello Via Paradisa, 2, 56124 Pisa, Italy; rosalinda.madonna@unipi.it; Tel.: +39-050-315-2683; Fax: +39-050-315-2684

The editorial refers to the Special Issue “Pulmonary Arterial Hypertension: Old Drugs and New Treatment Strategies”.

Pulmonary arterial hypertension (PAH) is a rare disease characterized by pulmonary vascular remodeling and elevated pulmonary pressure, causing right heart failure and death [1]. International registries have shown a female predominance of the disease, hypothesizing a hormonal involvement in its pathobiology [2]. The same registries also show a progressive increase in cardiovascular comorbidities (e.g., systemic hypertension, hyperlipidemia, obesity, diabetes mellitus, peripheral arterial disease, coronary artery disease) among patients with PAH, although it is not yet clear how they may affect the evolution of pulmonary vascular disease and the response to specific treatment [3].

Endothelial cells of different microvascular endothelial cells (ECs) participate in organogenesis and organ repair through the release of trophogens with paracrine activity called angiocrine factors (AFs) [4]. They are able to promote the organogenesis and repair of the parenchyma and adjacent stroma of organs where ECs themselves are allocated. It is possible that pulmonary microvascular cells play a key role in PAH through the release of specific Afs [4].

Genetic studies have shown that the bone morphogenetic protein type II receptor (BMPR2) plays a critical role in the pathogenesis of hereditary PAH [5]. Hereditary PAH accounts for at least 6% of all cases of PAH and has an autosomal-dominant mode of inheritance of the BMPR2 mutation. Heterozygous mutations of the BMPR2 gene have been found in approximately 50–70% of cases of hereditary PAH. The mutant allele is located in exons 4 and 5 of the BMPR2 gene, which encode the transmembrane domain and part of the kinase domain of BMPR2. Mice that are heterozygous for this mutant allele of BMPR2 (BMPR2+/− mice) survive and reproduce normally, whereas homozygous mutant mice die during gastrulation due to failure to specify the mesoderm. In pulmonary arterial smooth muscle cells isolated from BMPR2+/− mice, BMPR2 mRNA levels are reduced by 50% and the activation response of Smad1/5/8 to BMP2 and BMP7 is attenuated. Under resting/control (basal) conditions, BMPR2+/− mice exhibit mild pulmonary hypertension, with greater wall thickness of muscularized pulmonary arteries than wild-type mice. However, vascular lesions typical of patients with severe PAH, such as neointima formation or plexiform lesions, were not detected in the lungs of BMPR2+/− mice. These results suggest that additional genetic and/or environmental factors (such as comorbidities, infectious diseases, and cardiovascular risk factors) may cause more severe phenotypes in BMPR2+/− mice. BMPR2+/− mice would represent a useful preclinical model to examine the effects of risk factors and comorbidities on pulmonary vascular remodeling in hereditary PAH [5].

Correct diagnosis and proper clinical framing of pulmonary hypertension are prerequisites for appropriate treatment [6,7,8,9,10], which can improve not only symptoms and quality of life but also patient survival. The implementation of more aggressive therapy protocols, focused on the up-front combination of synergistically acting drugs, and the availability of new molecules acting on alternative cellular and molecular targets represent today’s crucial challenges on which research and care must focus efforts and resources. However, patients with PAH and comorbidities treated with initial combination therapy [11] showed a more attenuated treatment response.

This Special Issue aims to disseminate the latest knowledge in the diagnostic, prognostic, and therapeutic fields of the various conditions that have pulmonary hypertension as a common element.

As a Guest Editor, I thank the authors, reviewers, and support of the *JCM* team.

## References

[1] Humbert M., Kovacs G., Hoeper M.M., Badagliacca R., Berger R.M.F., Brida M., Carlsen J., Coats A.J.S., Escribano-Subias P., Ferrari P. (2022). 2022 ESC/ERS Guidelines for the diagnosis and treatment of pulmonary hypertension. Eur. Heart J..

[2] Cheron C., McBride S.A., Antigny F., Girerd B., Chouchana M., Chaumais M.C., Jaïs X., Bertoletti L., Sitbon O., Weatherald J. (2021). Sex and gender in pulmonary arterial hypertension. Eur. Respir. Rev..

[3] Panagiotidou E., Boutou A., Kalamara E., Sourla E., Chatzopoulos E., Stanopoulos I., Pitsiou G. (2021). Diagnosis and management of combined post- and precapillary pulmonary hypertension in a patient with multiple comorbidities. Adv. Respir. Med..

[4] Madonna R. (2022). Angiocrine endothelium: From physiology to atherosclerosis and cardiac repair. Vascul. Pharmacol..

[5] Ryanto G.R.T., Ikeda K., Miyagawa K., Tu L., Guignabert C., Humbert M., Fujiyama T., Yanagisawa M., Hirata K.I., Emoto N. (2021). An endothelial activin A-bone morphogenetic protein receptor type 2 link is overdriven in pulmonary hypertension. Nat. Commun..

[6] Madonna R., Fabiani S., Morganti R., Forniti A., Mazzola M., Menichetti F., De Caterina R. (2021). Exercise-induced pulmonary hypertension in HIV patients: Association with poor clinical and immunological status. Vascul. Pharmacol..

[7] Madonna R. (2021). Exploring the mechanisms of action of gliflozines in heart failure and possible implications in pulmonary hypertension. Vascul. Pharmacol..

[8] Mazzola M., Madonna R., Badagliacca R., Caterina R. (2022). Porto-pulmonary arterial hypertension: Translation of pathophysiologica concepts to the bedside. Vascul. Pharmacol..

[9] Madonna R., Morganti R., Radico F., Vitulli P., Mascellanti M., Amerio P., De Caterina R. (2020). Isolated Exercise-Induced Pulmonary Hypertension Associates with Higher Cardiovascular Risk in Scleroderma Patients. J. Clin. Med..

[10] Pugliese N.R., Mazzola M., Madonna R., Gargani L., De Biase N., Dini F.L., Taddei S., De Caterina R., Masi S. (2022). Exercise-induce pulmonary hypertension in HFpEF and HFrEF: Different pathophysiologic mechanism behind similar functional impairment. Vascul. Pharmacol..

[11] McLaughlin V.V., Vachiery J.L., Oudiz R.J., Rosenkranz S., Galiè N., Barberà J.A., Frost A.E., Ghofrani H.A., Peacock A.J., Simonneau G. (2019). Patients with pulmonary arterial hypertension with and without cardiovascular risk factors: Results from the AMBITION trial. J. Heart Lung Transplant..

